# Seasonal variation in a diverse beetle assemblage along two elevational gradients in the Australian Wet Tropics

**DOI:** 10.1038/s41598-018-26216-8

**Published:** 2018-06-04

**Authors:** C. W. Wardhaugh, M. J. Stone, N. E. Stork

**Affiliations:** 1 Faculty of Science, University of South Bohemia, Branišovska 31, 370 05 Ceske Budejovice, Czech Republic; 2 Environmental Futures Research Institute, School of Environment and Science, Griffith University, Nathan Campus 170 Kessels Road, Nathan, Queensland 4111 Australia; 3 Present Address: Scion (New Zealand Forest Research Institute), PO Box 3020, Rotorua, 3010 New Zealand

## Abstract

Altered abiotic conditions resulting from human-induced climate change are already driving changes in the spatial and temporal distributions of many organisms. For insects, how species are distributed across elevations is relatively well known, but data on their seasonality at different elevations are lacking. Here we show seasonal variation in beetle abundance and species richness along two spatially-distinct elevational transects (350–1000 m and 100–1000 m asl) in the rainforests of northern Australia. Temperature was the best predictor of temporal abundance and species richness patterns, while rainfall had little influence. Elevation had little effect on seasonal changes in abundance or diversity. Adults of most beetle species exhibited long season-lengths (>6 months of the year) with distinct peaks in abundance during the summer wet-season. We found evidence of phenotypic variation among the more widespread species, with seasonal peaks in abundance often not coinciding across elevations or transects. Due to the wide elevational range of most species, and the lack of consistency in the seasonality of wide-spread individual species, we suggest that many beetles inhabiting the low to mid-elevation mountains in the Wet Tropics, and potentially other tropical rainforests, are not as vulnerable to extinction due to climate change as many other organisms.

## Introduction

There is increasing concern that climate change will result or is already resulting in latitudinal and/or elevational shifts in the distribution of many organisms^1,2^. Some authors suggest that terrestrial tropical communities will be particularly adversely impacted by climate change due to a high incidence of elevational-specialist species, and reduced latitudinal climatic gradients that could facilitate range shifts^1,3–5^. Consequently, increasing temperatures are predicted to result in tropical species having to range-shift up mountains in order to remain within climatically tolerable conditions^3^. For faunal groups that exhibit high-elevation range restrictions, such as many vertebrates and flightless beetles, climate change is predicted to exacerbate the extinction rate^1,3,6^. However, although numerous studies examine how spatial distributions of abundance and diversity change with respect to elevation^7–9^, very few simultaneously examine temporal changes in assemblage dynamics at different elevations^10–12^.

Temporal patterns of abundance and diversity that reflect changing seasonal climatic conditions are frequently ignored in tropical rainforests^13^. Patterns of seasonality among invertebrates in particular are poorly understood, with a few notable exceptions^11,12,14–20^. Wolda^15^ used a 14 year dataset of light-trapped insects in Panama to demonstrate that populations of insects change both within and between years. In tropical Australia, Frith and Frith^16,17^ showed that insect abundance correlated with seasonal changes in rainfall and moisture availability. The importance of moisture availability to seasonality has also been demonstrated for sap-sucking insects in Papua New Guinea^21^, herbivorous insects in Panama^22^ and Guyana^23^, beetles in Uganda^24^, geometrid moths in Peninsular Malaysia^25^, and scarab beetles in Borneo^26^. With climate change predicted to alter rainfall patterns, the moisture-dependent seasonality of tropical insects is certain to change, but the effect of climate change on tropical insect seasonality remains poorly understood.

Many arthropods have mechanisms to reduce the risk of death or population crashes during periods when abiotic conditions are unfavourable. Some species enter a state of low activity or dormancy (diapause) at the egg, pupal or adult stages^27–29^. Insect species may also display a range of biological and behavioural changes to avoid seasonably harsh climatic conditions. These include migrating^10,30,31^, and aggregating with others of the same species in micro-refugia that experience more favourable conditions^28^. Alternatively, the abundances of species may simply rise and fall with temporal changes in resource availability. For example, Southwood *et al*.^32^ and Stork & Hammond^33^ showed how temporal patterns in the abundances of species or feeding guilds of beetles were linked to the timing of leaf flush and flower availability in European oak trees. While the seasonality of some species may be rigid (perhaps triggered by fixed factors like photoperiod), many species also exhibit substantial plasticity in their responses to climatic variability, allowing them to survive unpredictably harsh conditions. These physiological responses to unfavourable climatic conditions are invariably triggered by changes in abiotic conditions, primarily temperature or moisture availability^27,28^.

With a few notable exceptions^10–12,20,34,35^, there are a lack of studies that explicitly incorporate both temporal and elevational aspects in an examination of insect abundance and diversity patterns. In this paper we examine seasonal changes in the abundances of beetles at different elevations in the Wet Tropics rainforests of North Queensland, Australia. We chose to study beetles as a representative group of insects since these number roughly a quarter of an estimated 5.5 million species of insects on Earth^36^, are the most trophically diverse of all insect orders, and have ancient lineages^37^. The aim of this study was to identify seasonal peaks in beetle abundance and diversity and ascertain if these vary with elevation, and whether that variation correlates with climatic variation (temperature or rainfall) among elevations. We hypothesise that the magnitude of seasonality in beetle abundance and diversity will increase with increasing elevation, resulting in near-aseasonal lowlands and highly seasonal high-lands, due to greater seasonal variation in abiotic conditions, particularly temperature, at higher elevations. By contrast, similar seasonality patterns across elevations may indicate that beetles are insensitive to the variation in abiotic conditions experienced on these mountains. Whereas inconsistent or random seasonality patterns across elevations may indicate a high level of plasticity in temporal activity patterns within species.

## Methods

### Site survey

**Figure 1:**
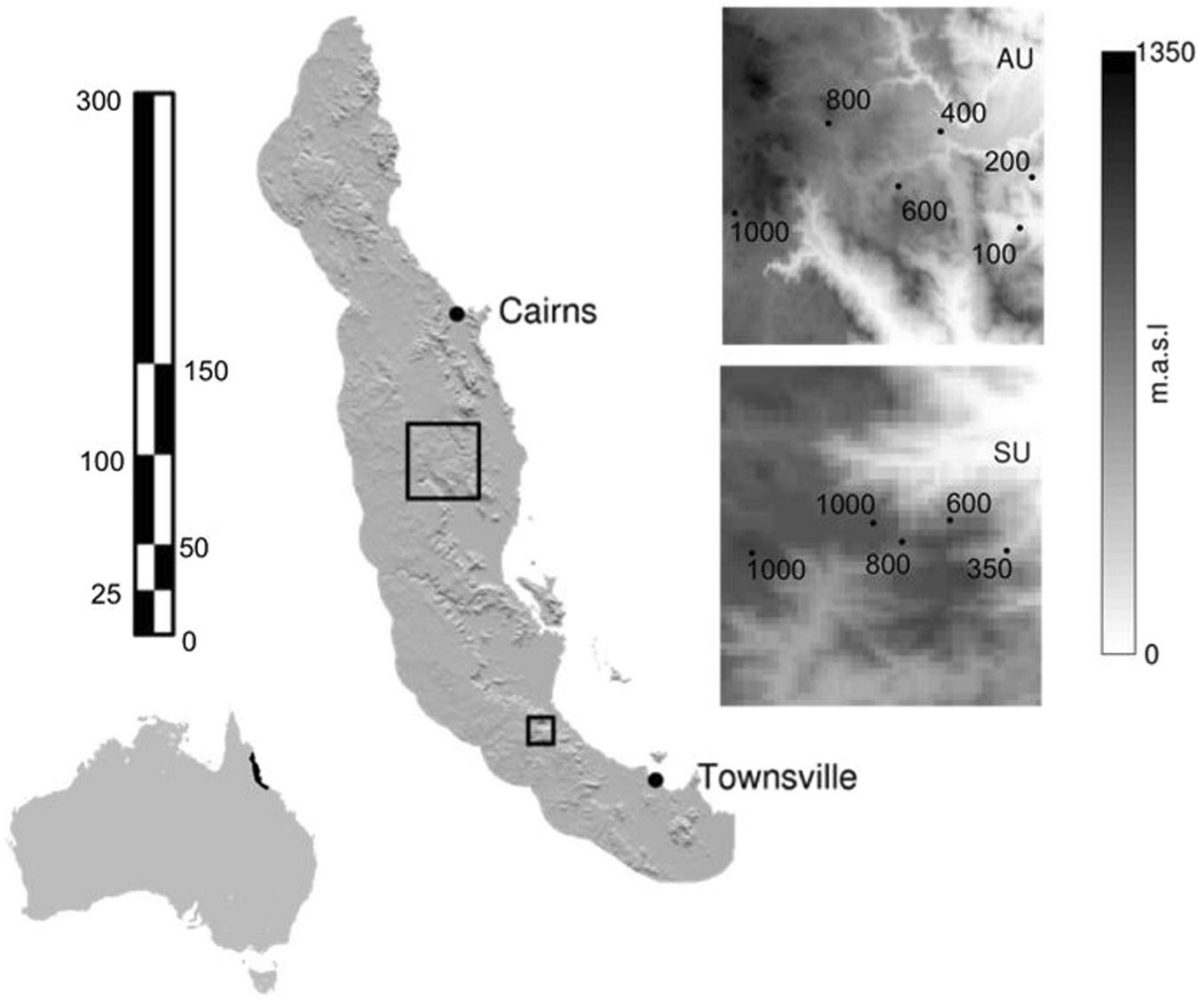
Map showing the Wet Tropics region and the location of the two transect areas (with left hand scale in Kms). The inset maps show the locations of the sample sites (with right hand scale showing shading to represent m.a.s.l.) on the Atherton transect (AU = Atherton Uplands) and the Paluma transect (SU = Spec Uplands after Mt Spec in Paluma). Figure created using the package SDMTools in R (GEODATA 9 S DEMVersion 2; © Commonwealth of Australia (Geoscience Australia) 2017, http://www.ga.gov.au/)^43,50^.

The Wet Tropics World Heritage Area of Queensland is the last and largest remaining northern remnant of what was (~30 mya) an extensive rainforest covering large parts of Australia. It is dominated by a north-south mountainous region (part of the Great Dividing Range), which rises rapidly from the coastline bordering the Great Barrier Reef in the east to a maximum of 1,622 m asl (Mt Bartle Frere) in the west^38^. Our study comprised two elevational transects on the eastern slopes, approximately 200 km apart, in the southern and central parts of the Wet Tropics (Fig. 1). In the southern transect (Paluma - Mt Spec Uplands, hereafter referred to as Paluma), sites were established at 350, 600, 800 m and two at 1,000 m asl. Mt Spec itself rises to an elevation of little more than 1,000 m asl, so our transect covered most of the available elevational range. The central transect (Atherton Uplands) included rainforest sites at 100, 200, 400, 600, 800, and 1,000 m asl). The highest point in the Atherton uplands is Mt Fisher at 1,385 m asl. This transect therefore, covered about three quarters of the elevational range of the Atherton Uplands.

Rainforest types were categorised as notophyll vine forest (Paluma 350, 600, 800 m, Atherton 800, 1,000 m), complex mesophyll vine forest (Atherton 100, 200, 400, 600 m), and closed *Acacia* forest (Paluma 1,000 m). All sites had closed canopies of more than 90% foliage cover. Mean annual rainfall (1990–2014) on the Paluma transect increased slightly with elevation from 1,519 mm at 350 m to 1,840 mm at 1,000 m, while mean annual rainfall decreased with increasing elevation on the Atherton transect from >3,200 mm at 100 and 200 m to 1,602 mm at 1,000 m (for more details see Table S1). Temperature data (daily maximum and minimum) for each site were obtained from weather stations located within 20 m of our insect traps. Temperature data were collected hourly. Daily rainfall totals were collected at Bureau of Meteorology (BOM) weather stations distributed through the region. On the Paluma transect, only three BOM weather stations serviced all five sites due to the geographical (if not elevational) proximity of several sites. The 350 m and 600 m site rely on the same rainfall data, while the 800 m site and 1,000 m(b) also share a single BOM weather station. On the Atherton transect, rainfall data for each elevation was obtained from unique BOM weather stations.

### Beetle sampling

Beetles were sampled with ground-based flight interception traps (FIT). These traps were a modified Malaise trap with collecting containers positioned along the bottom edge and filled with a solution of propylene glycol as a killing and preserving solution. This trap design was successfully used in previous studies in this region to quantify seasonality patterns among rainforest beetle assemblages^18^. It should be noted that FITs do not measure true abundances of insects, but rather they measure activity-density (i.e. individuals collected per trap per day). Despite losing a measure of spatial variability, we chose to use a single trap at each elevational site for three reasons. First and foremost, these traps are remarkably consistent in their catch compared to other kinds of traps^39^, so the use of single traps is unlikely to result in biased capture rates or inaccurate diversity measures. Second, these traps capture very high abundances of beetles, which introduces a logistical sorting problem when many traps are used over long periods of time. And thirdly, since site access was often problematic, pseudo-replication would likely have become an important issue due to the inevitable close proximity of multiple traps at each site.

FITs were continuously operated between December 2006 and December 2007. Access to sites was sometimes restricted due to weather and hence the contents of traps were collected at 9 (Atherton) or 10 (Paluma) irregular intervals that ranged from a minimum of 24 days to a maximum of 59 and 53 days respectively for the Atherton and Paluma transects. Two samples (December-February 2006–7) from the 600 m site along the Atherton transect were not collected due to problems with site access. The 600 m site therefore, has been omitted from analyses involving the calculation of seasonality, but included in figures showing seasonal patterns in abundance and diversity. A further two samples (Atherton 400 m, August 29th–October 25th; and Paluma 600 m, August 21st–September 25th) were lost during fieldwork.

Because of the very large number of individual beetles encountered (see results) only a subset of beetle families were sorted to species. Seven beetle families or sub-families (Carabidae, Histeridae, Staphylinidae: Pselaphinae, Laemophloeidae, Zopheridae, Chrysomelidae, and Curculionidae: Scolytinae), were dry-mounted and sorted to species or morphospecies by comparing against an extensive reference collection assembled from specimens collected from the Atherton Tablelands in previous studies^40,41^. These particular beetle families were selected because they collectively represent the ecological diversity of this large insect Order, they had been the focus of previous studies in the Wet Tropics, and were well represented in available reference collections. Each family selected represents a different superfamily of beetles, within three different series, and two of the four Suborders of Coleoptera.

### Data analysis

Seasonal variation in beetle activity-density (hereafter referred to as abundance) and species diversity was standardised for subsequent analysis to the numbers of individuals and species captured per trap day to account for uneven temporal sampling periods. Analysis of seasonal changes in abundance/trap day (Atherton; N = 8 to 9 repeat samples, Paluma; N = 8 to 9 repeat samples) were carried out for all beetles and separately for each of the seven focal beetle families, at both transects and each elevation (Atherton; N = 6 elevations, Paluma; N = 4 elevations). Patterns in species diversity/trap day at each transect and elevation were restricted to beetles within the seven focal families. Species diversity/trap day was chosen over standard rarefaction for two reasons. First, the traps we used are not affected by previous sampling success, so standardising species-diversity by the abundance of collected individuals is not necessary. Second, rarefaction is sensitive to high abundances, where one or two very common species can drive down rarefied species diversity, giving a false impression of species diversity patterns.

We used nlme^42^ in R^43^ to perform linear mixed effect (LME) analyses to create random intercepts models using the ‘lme’ function of the effect of season, elevation and climatic variables of rainfall and maximum and minimum temperature on abundance and species diversity. Seasons were defined as follows: early wet season, or summer (Dec-Feb), late wet season, or autumn (Mar-May), early dry season, or winter (Jun-Aug), and late dry season, or spring (Sep-Nov). In the full models, elevation (N = 5), season (N = 4) and the climatic variables were fixed and site group was the random effect to control for repeated measures. The sample N for the Atherton transect in the LME analyses was reduced to five because the 600 m elevation was excluded due to missing samples during the middle of the raining season (Jan-Dec). Models were reduced and compared and chosen for best fit using both AIC by REML and P-values obtained by Maximum Likelihood ratio tests. Models selected for each beetle attribute of total abundance, abundance of the seven focal families and species diversity were reduced to test the contribution of each of the fixed factors to the full model. Post-hoc Tukey tests with Bonferroni-Holm corrections in season and elevation pair-wise multiple comparisons were made with the ‘glht’ function in the package multcomp^44^.

The temporal distribution of beetle species was first measured by the parameter season-length (SL)^45^, which provides an indication of the minimum tolerance of individual species to prevailing biotic or abiotic conditions. SL is defined as the total number of temporal periods minus the longest sequence of sampling periods where that species was not recorded, thus ranging from 1 (all individuals collected in one sample) to 9 at Atherton or 10 at Paluma. This measure was calculated for beetle species on each transect, and separately for each elevation on each transect to ascertain whether SL varied between transects and elevations. Individual species that occurred at more than one elevation or on both transects could be analysed more than once.

Most tropical insect species are present as adults for an extended proportion of the year, but show marked changes in abundance through time^14,21^. Season-length, which provides presence/absence information on seasonality, therefore may not be suitable for detecting seasonality in abundance among our samples. We consequently used circular statistics (Rayleigh’s test of circular uniformity)^46^ in Oriana (version 4) to detect significant temporal variation in the abundance of each beetle species. In this analysis, temporal periods were expressed as vectors corresponding to the days of the year on which sampling occurred. This test provided three pieces of information. First was a statistic that tested the null hypothesis of seasonal uniformity in abundance, allowing us to categorise each species as seasonal or aseasonal. The second was the mean vector, which when transformed back to days of the year reports when each species reached its peak abundance. And the third was a measure of the degree of seasonality, called the length of the mean vector (LMV)^46^. The LMV is a measure of how closely observations are clustered around the mean, and ranges from 0 (perfect temporal uniformity) to 1 (all individuals collected during one sampling period). Variation between transects and elevations in mean SL and LMV of beetle assemblages were investigated with ANOVA and posthoc Tukey tests.

### Data availability statement

The datasets generated during and/or analysed during the current study are available from the corresponding author on reasonable request.

## Results

### Seasonal variation in abundance

Between December 2006 and December 2007, a total of 267,657 beetles were sampled from the six elevational sites along the Atherton transect, and 50,862 beetles were sampled from the five sites at four elevations along the Paluma transect. The LME model selection process indicated that the best model fit for total abundance and abundance of the seven focal families should include season for both the Atherton and Paluma transects (Table S2). Despite this, season was only significant at the Paluma transect in the season reduced models for total abundance (AIC = 450, P = 0.03) and abundance of the seven focal families (AIC = 169, P = 0.02) (Table 1, Fig. S1). However, post-hoc Tukey pair-wise tests did not indicate a significant difference between seasons (Table S3).

**Table 1:** Reduced LME models of total abundance, abundance of the seven focal families and species diversity of the seven focal families.

Transect	Fixed variable reducted	Beetle attribute	AIC^d^	L.ratio	*df*	*P*
Atherton	Season	Abundance^a^	468	0.21	10	0.98
		Abundance^b^	190	3.20	8	0.07
		Spp. diversity^c^	42	7.08	8	0.07
	Elevation	Abundance^**a**^	**490**	**21.1**	**9**	**0.0003**
		**Abundance** ^b^	**188**	**11.3**	**7**	**0.02**
		Spp. diversity^**c**^	**45** ^e^	**18.4**	**7**	**0.001**
	Rainfall	Abundance^a^	447	0.5	12	0.49
	Tmin	Abundance^a^	452	0.25	11	0.62
		**Spp. diversity** ^c^	**72**	**38.9**	**10**	**<0.0001**
	Tmax	Abundance^a^	452	0.47	12	0.49
		Abundance^**b**^	**187**	**12.3**	**10**	**0.0005**
Paluma	Season	Abundance^**a**^	**450**	**9.03**	**9**	**0.03**
		**Abundance** ^b^	**169**	**9.87**	**7**	**0.02**
		Spp. diversity^c^	**38**	**19.4**	**7**	**0.0002**
	Elevation	**Abundance** ^a^	**450**	**12.2**	**9**	**0.007**
		**Abundance** ^b^	**166**	**7.96**	**7**	**0.05**
		**Spp. diversity** ^c^	**28** ^e^	**10.7**	**7**	**0.01**
	Rainfall	Abundance^a^	428	3.93	11	0.05
	Tmin	Abundance^a^	429	0.43	11	0.51
		Spp. diversity^c^	**60**	**37.6**	**9**	**<0.0001**
	Tmax	Abundance^a^	430	0.89	11	0.35
		Abundance^**b**^	**174**	**15.6**	**9**	**0.0001**

Each fixed variable has been individually reduced from the full model (full models shown in Table S2) and results from both AIC in REML and by Maximum Likelihood are shown. Significant variables are shown in bold.

^a^Total abundance (mean per trap day) full model: lme(TotalAbund ~ Elevation + Season + Rainfall + Tmax + Tmin,random = ~1|Site.group).

^b^Abundance of the seven focal families (mean per day) full model: lme(FamAbund~Elevation + Season + Tmax,random = ~1|Site.group).

^c^Species diversity of the seven focal abundant families (mean per day) full model: lme(Spp.diversity ~ Elevation + Season + Tmin,random = ~1|Site.group).

^d^AIC’s of the reduced models and fit by REML.

^e^AIC’s are lower than the full model AIC shown in Table S2.

Elevation may be a better predictor of beetle abundance than season because models reduced by elevation show that elevation had a significant effect in both transects on total abundance (Atherton: AIC = 490, P < 0.0001, Paluma: AIC = 450, P = 0.03) and on abundance of the seven focal families (Atherton: AIC = 188, P = 0.02, Paluma: AIC = 166, P = 0.05) (Table 1). Although, post-hoc Tukey tests revealed significant differences between elevations at Paluma only. These differences at the Paluma transect were for total abundance between 350 m and 1,000 m (z = 3.89, P = 0.0006) and in abundance of the seven focal families between 350 m and 1,000 m (z = 3.93, P = 0.0005) and between 600 m and 1,000 m (z = 2.61, P = 0.04) (Table S3; Fig. S1).

**Figure 2:**
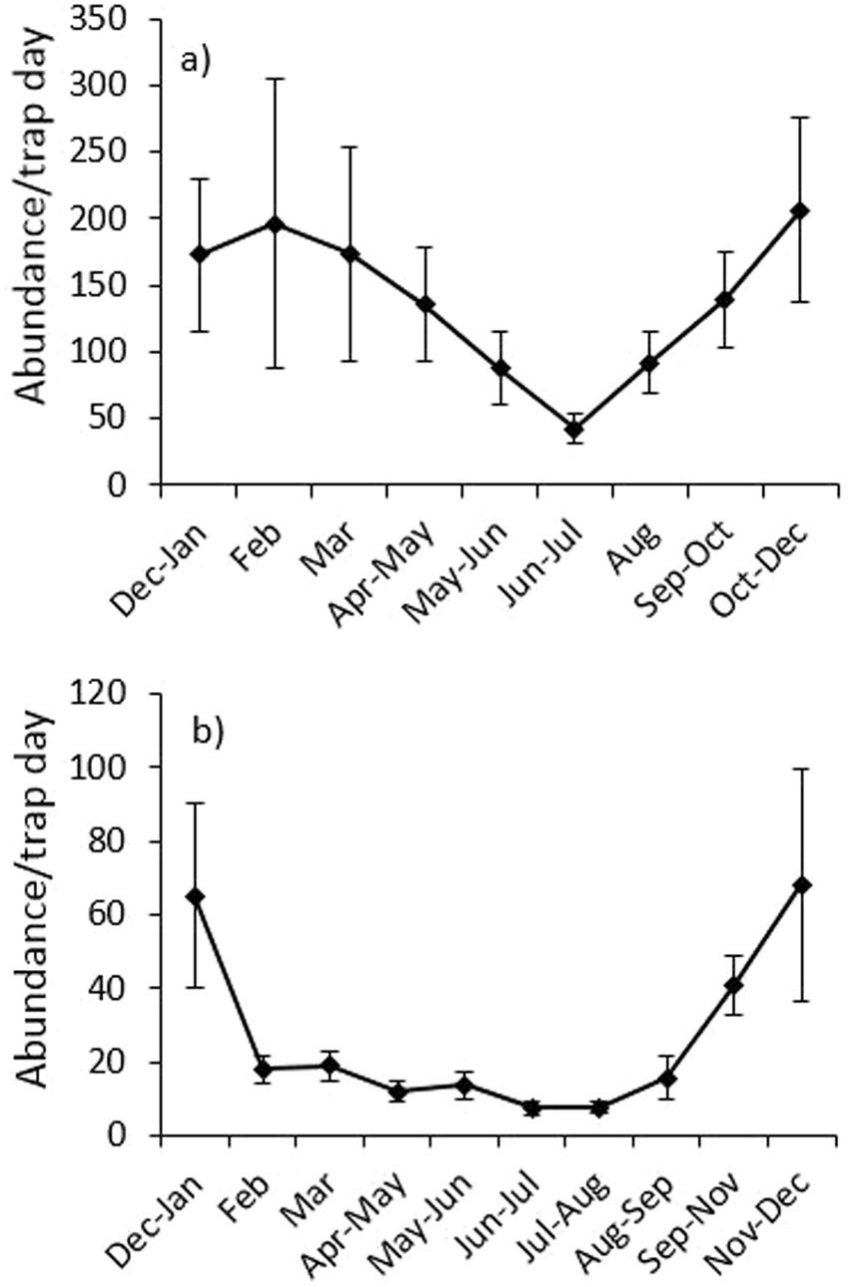
Seasonal variation in the mean (±SE) abundance/trap day of all beetles across all elevations on (**a**) the Atherton transect, and (**b**) the Paluma transect. Note that the temporal axes are not to scale.

**Figure 3:**
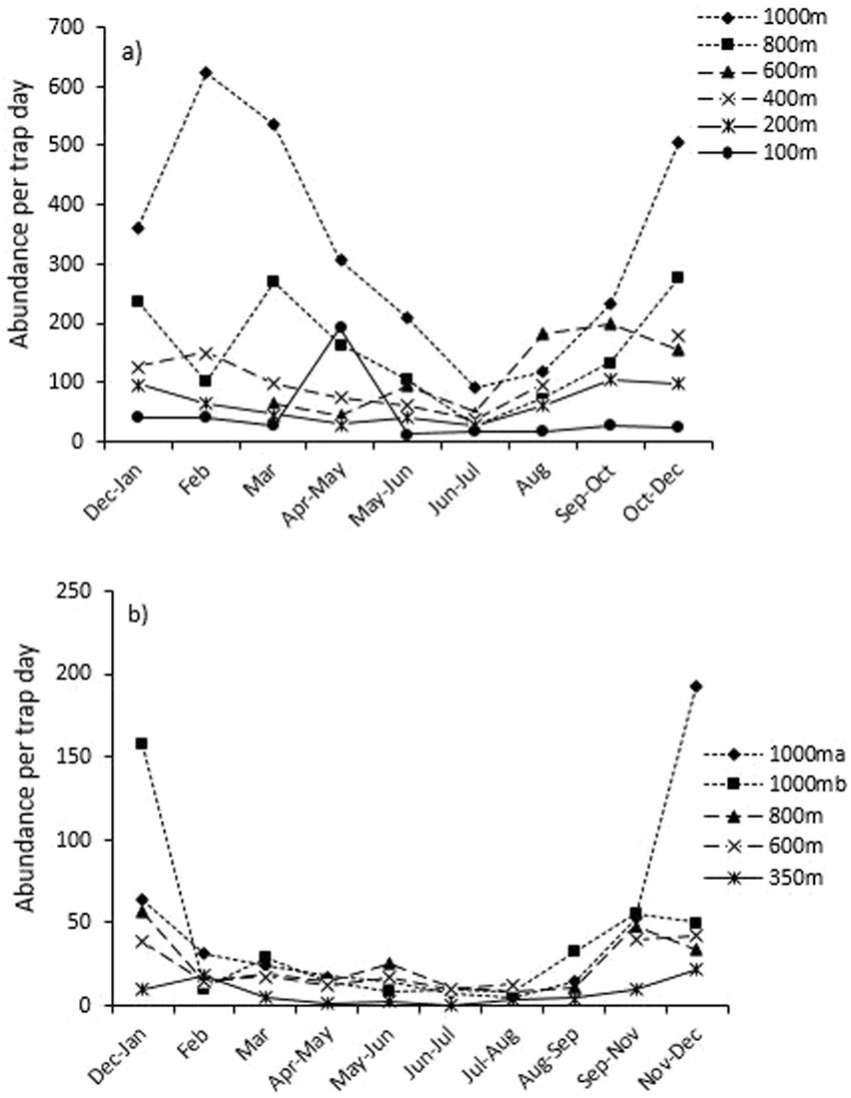
Seasonal variation in the abundance of beetles sampled from flight interception traps at different sites on the Atherton transect (**a**) and the Paluma transect (**b**) in Queensland’s Wet Tropics between December 2006 and January 2008. Samples were standardised to numbers of beetles per trap day to account for slightly uneven temporal sampling periods. Note that the temporal axes are not to scale.

Despite a lack of effect between seasons in abundance using post-hoc Tukey tests (in which grouped data was necessary for this analyses approach, hence diluting the strength of seasonal effect), season was still an important contributor to the full model (Table 1). Plus clear differences in abundance can be seen through different times of the year at both Atherton and Paluma in Figs 2 and 3 and S1, as well as for species richness within each of the seven focal families in Figs S2 and S3. The seasonal peak in the total abundance of beetles was generally in the hottest months (Sept.-May at Atherton and Sept.-Jan. at Paluma; Fig. 2). This pattern was consistent across all elevations on both transects (Fig. 3), where abundances peaked between September and May, and reached their lowest ebb in the cool winter months (June-July; Fig. 3). Abundance patterns for each elevational site and beetle family were either consistent with these larger scale patterns, or were erratic and showed no clear seasonal pattern (Figs S2 and S3). Summer peaks in abundance appeared to be more marked at higher elevations (800 and 1,000 m at Atherton, 1,000 m at Paluma; Fig. 3).

### Seasonal variation in species diversity

A total of 17,069 individuals from 342 species at Atherton, and 3,128 individuals from 247 species at Paluma were collected from the seven beetle families and subfamilies chosen for analyses of species diversity patterns.

The LME model selection process indicated that the best fit model for species diversity of the seven focal families should include season for both the Atherton and Paluma transects (Table S2). However, post-hoc Tukey tests revealed no difference in species diversity between seasons at either transect (Table S3). The greatest effect on species diversity was elevation at both Atherton (AIC = 45, P = 0.001) and Paluma (AIC = 28, 0.01) (Table 1). But this was only the case when considering p-values only, because the AIC’s were lower than each transects respective full models (Tables 1 and S2). Irrespective of lower AIC’s in the reduced models, differences in species diversity for season were detected in post-hoc Tukey’s pair-wise tests. For Atherton these were between 1,000 m and 100 m (z = 4.22, P = 0.0002), 1,000 m and 200 m (z = 3.0, P = 0.02), 800 m and 100 m (z = 5.15, P < 0.0001) and between 800 m and 200 m (z = 0.001). At Paluma the differences were only between 1,000 m and 350 m (z = 3.35, P = 0.005) (Table S3).

**Figure 4:**
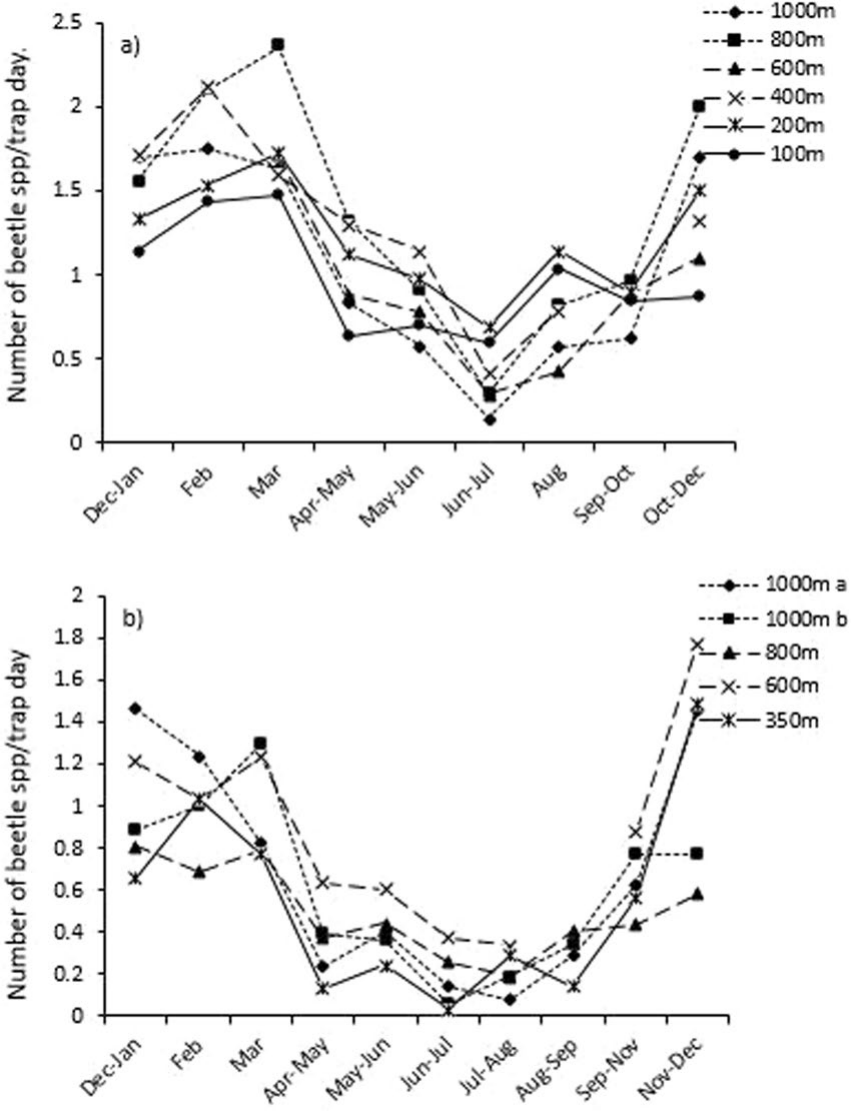
The number of beetle species collected per trap day at each elevation on (**a**) the Atherton transect, and (**b**) the Paluma transect. Note that the temporal axes are not to scale.

**Figure 5:**
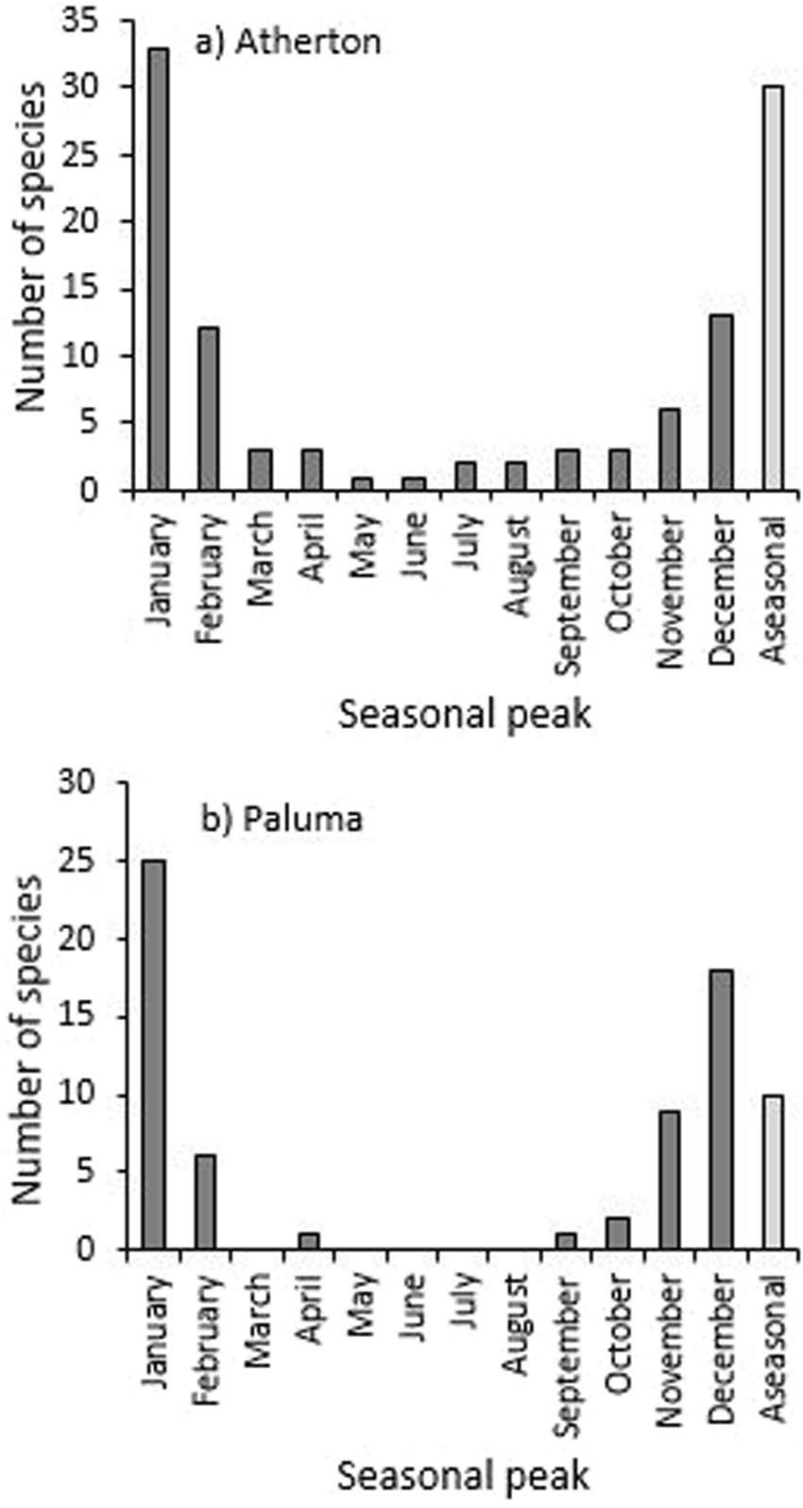
The number of significantly seasonal species (as indicated by Rayleigh’s test of circular uniformity) reaching their seasonal peak (mean vector) in abundance during each month at (**a**) Atherton and (**b**) Paluma. Also shown are the number of aseasonal species whose temporal abundance distributions did not differ from uniformity.

Despite a lack of effect between seasons in species richness in post-hoc Tukey tests, similarly for abundance, season was still an important contributor to the full model (Table 1). Clear differences in species richness could also be seen through different times of the year at both Atherton and Paluma in Fig. 4 and Figs S1 and S4, and for species richness within each of the focal seven families in Figs S5 and S6. Here the seasonal patterns in species diversity followed that of beetle abundance, where the number of species/trap day was highest in the warmer months (September/October to March), and lowest in the cooler dry-season (June-August; Fig. S4). Again, this pattern was consistent across elevations, where species diversity was highest between October and March at every elevation, and lowest in June/July for every site on the Atherton transect (Fig. 4a). The species richness of individual families at each elevation on the Atherton transect also showed similar patterns to those in Fig. 3a (Fig. S5). On the Paluma transect, species diversity peaked at every elevation between November and March, while it was lowest between June and September for each elevation (Fig. 4b). Individual families also largely followed this trend (Fig. S6). The frequency distribution of the mean vector for each beetle species also showed that most seasonal species reached peak abundance during the summer wet-season (December/January) on both transects (Fig. 5). This was also the case when the distribution of mean vectors was examined for each elevation and individual family on each transect (data not shown).

### Seasonality of beetle species

**Figure 6:**
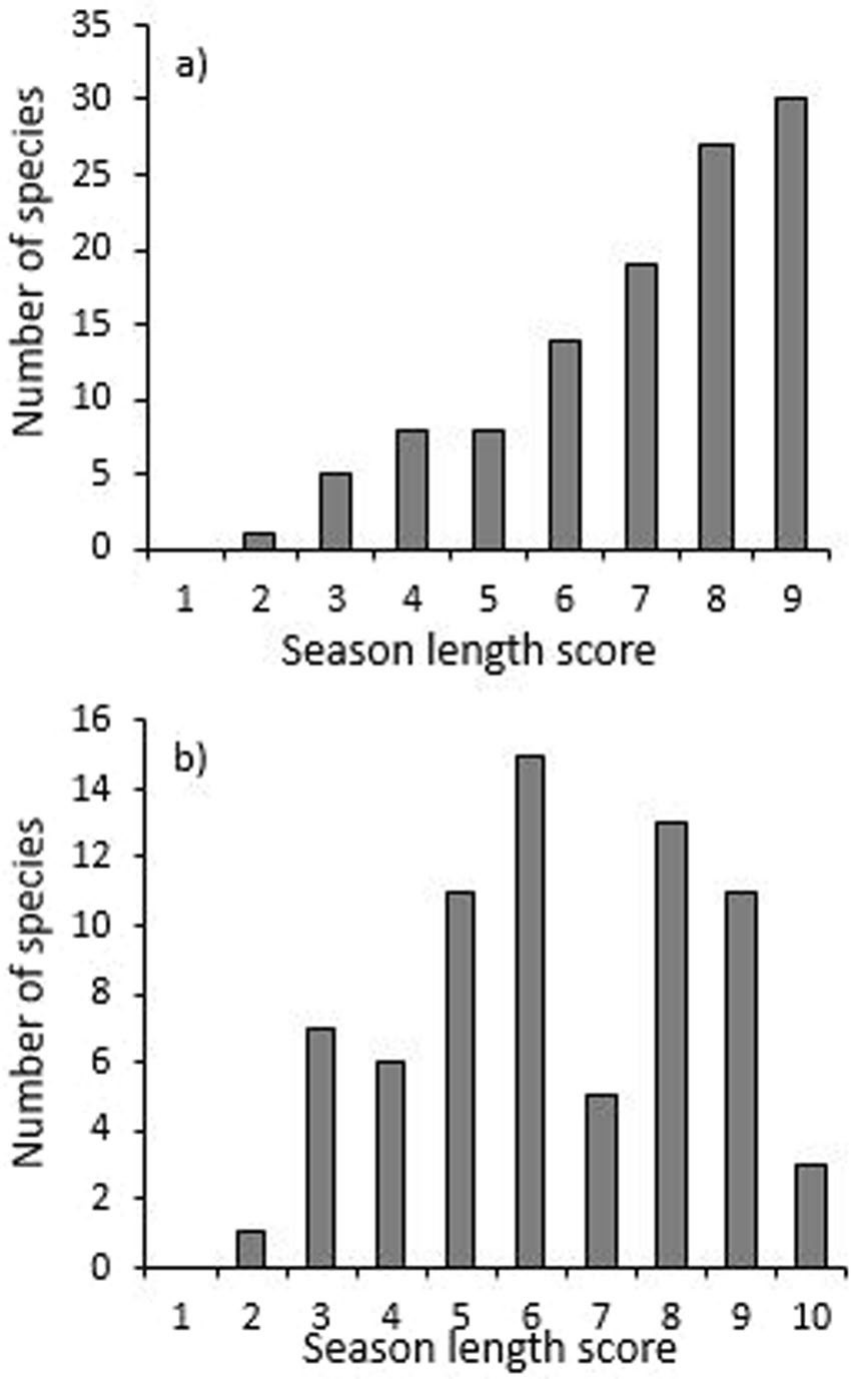
The frequency distribution of season-length scores across beetle species where n ≥ 10 individuals for (**a**) the Atherton transect, and (**b**) the Paluma transect.

There were 112 species on the Atherton transect, and 72 species on the Paluma transect with an abundance of at least 10 individuals that were included in species-level analyses. Most species had relatively long season-lengths, particularly on the Atherton transect where just 22/112 species (19.6%) were recorded over a period of six months or less (Fig. 6a). On the Paluma transect, a greater proportion of species (25/72, or 34.7%) were restricted to six months or less of the year (Fig. 6b). Patterns in SL were also consistent among elevations and for individual families on each transect (data not shown), where most species displayed long season-lengths. Species from Paluma exhibited significantly shorter season-lengths (F_1,182_ = 5.492, P = 0.020) than species from Atherton (mean SL = 6.389 and 7.071 respectively), despite the fact that there was an extra temporal sample collected from Paluma, which would have slightly biased this metric toward greater SL on that transect. There was no significant variation across beetle families in season-length (F_6,177_ = 2.059, P = 0.060), nor were there any significant differences in SL between families on different transects (F_12,171_ = 1.515, P = 0.123).

Rayleigh’s test showed that most species on both transects experienced strong seasonality in abundances (at Atherton 82/112 (73.2%) species, and at Paluma 62/72 (86.1%) were significantly seasonal; Fig. 5). An ANOVA examining mean LMV between transects showed that beetles from Paluma (mean LMV = 0.632) were significantly more seasonal in abundance (F_1,182_ = 16.908, P < 0.0001) than beetles from Atherton (mean LMV = 0.495). This was also the case when analyses were restricted to the 36 species that were common to both transects, where mean LMV at Paluma (0.604) was significantly higher (F_1,70_ = 7.434, P = 0.008) than mean LMV at Atherton (0.475). Surprisingly, the mean vector of shared species (i.e., seasonal peak in abundance) did not correlate between the two transects (r = 0.125, P = 0.468), suggesting that the seasonality of species is not fixed. Indeed, only 6/36 (16.7%) species common to both transects reached their seasonal peak within the same month, and a further 20 (55.6%) peaked within the same three-month period (Table S4). Nine species (25%) were aseasonal on one transect, and seasonal on the other.

There was no difference in LMV between species from different families (F_6,177_ = 1.095, P = 0.367). There was however, significant variation in LMV between families on different transects (F_12,171_ = 2.316, P = 0.009), where posthoc Tukey tests revealed that pselaphines from Paluma were significantly more seasonal than pselaphines and laemophloeids from Atherton. There was no difference in the LMV of beetle species between elevations on the Atherton transect (F_5,166_ = 1.734, P = 0.13). However, LMV did vary significantly between sites on the Paluma transect (F_4,74_ = 3.258, P = 0.016). Posthoc Tukey tests showed that beetles from the 800 m site were significantly less seasonal than beetles from the 600 m site and one of the 1000 m (a) sites.

There were 58 species where at least 10 individuals were collected from more than one site, allowing for an examination of variation in seasonality within species at different elevations and transects. Four species (6.9%) were aseasonal at every site, while 25 species (43.1%) were aseasonal at one site at least. Of the remaining 29 species, 18 (31.0%) reached seasonal peaks at roughly the same time of year (within a three-month period) across all elevations and transects (Table S4). Only four species (6.9%) had a separation of more than five months between seasonal peaks at different sites. Of these, two are likely the consequence of the inclusion of the Atherton 600 m site, which did not include sampling from December to February when most species reached their seasonal peaks at other sites. Of the remaining two, one seems to be inexplicable, while the other appears to be a seasonal elevational migrant. In fact, there is evidence for elevational migrations for two species; Carabidae 5 and Carabidae 14. Both reach a significant seasonal peak in abundance at the 200 m site on the Atherton transect during the winter dry-season (May and August respectively), then also reach a seasonal peak at the 1,000 m site during the summer (January and February respectively).

### Relationships with climatic variables

The climatic variables of rainfall and maximum and minimum temperature variously contributed to the fit of models of total abundance, abundance of the seven focal families, and species richness (Tables 1 and S2). Contrary to many previous studies^16,17,21–23,25,26^, seasonal patterns in rainfall was not the best predictor of abundance and species richness of beetles and correlated poorly with all elevations on both transects (Tables 1 and S5).

On the Atherton transect, the LME models indicated that a combination of rainfall, and minimum and maximum temperature enhanced the model fits for total abundance (AIC = 445) (Table S2). When Tmin, Tmax, and rainfall were individually reduced from the full model, however, none were significant (Table 1). However, for species diversity the LME models indicated that minimum temperature enhanced the model fit (AIC = 49) which was strongly significant when reduced from the full model (AIC = 72, P < 0.0001) (Tables 1 and S2).

On the Paluma transect, the LME models indicated that a combination of rainfall, and minimum and maximum temperature enhanced the model fits for total abundance (AIC = 426) (Table S2). However, when each climatic variable was individually reduced from the full model, Tmin, Tmax were not significant and rainfall was only marginally significant (AIC = 428, P = 0.05) (Table 1).

## Discussion

Beetle assemblages in the tropical rainforests of north Queensland showed strong temporal variation, with total abundance and species diversity peaking in the summer wet-season (October to March) and reaching a low ebb in the winter dry-season (June to August). The strongest climatic variables to explain total abundance and species diversity patterns were temperature. Although rainfall was also a significant contributing variable in the full LME model for total abundance Contrary to expectation, we found little consistency between elevations in the timing of seasonal peaks in abundance or diversity, with many species showing marked seasonality at some sites and no seasonality at others. This indicates that the abundances of most species are not strictly controlled by single abiotic factors, since temporal patterns in rainfall and temperature were very similar across sites. Rather, the lack of widespread or consistent patterns in temporal changes in the abundances of beetle species indicates that temperature and rainfall patterns may have little effect on most beetle species in our study, with strong relationships between abiotic variables and beetle abundance only evident at the community level.

The degree of seasonality displayed by the beetle assemblages in our study did not vary consistently with elevation. Although seasonal changes in total beetle abundance were more marked for the 1,000 m site on the Atherton transect, and one of the 1,000 m sites at Paluma, there were no clear differences in abundance patterns among the other sites, or for species diversity. Furthermore, the greater seasonal changes in abundance seen at the highest elevations were the result of much higher abundances during the summer, rather than lower abundances during the winter, which was our expectation.

One possible explanation for the lack of strong elevational patterns in our study is that the mountains of Australia’s Wet Tropics are not high enough for changes in climatic variables to be great enough to significantly affect seasonality patterns among resident beetles. Indeed, mean temperature only varied by 3–4 °C, and rainfall patterns and dry-season days without rain were very similar, across all elevations on individual transects. This consistency in abiotic conditions along the entire elevational ranges of each of our transects may at least partly explain why we observed similar seasonal abundance and diversity patterns at most elevations. It may be more accurate then to describe the rainforests of north Queensland as lowland rainforest, perhaps grading into mid-elevation forest towards the highest elevations. Indeed, on a 2,200 m high transect in Panama, Wolda^7^ showed that insect abundance decreased gradually with increasing elevation, with some groups (including Chrysomelidae) only beginning to decrease in abundance above 1,000 m. Interestingly, the study of Boulter *et al*.^34^ who found similar results to us (a weak effect of elevation on abundance and diversity) was also limited to just 1,100 m in elevation. It is feasible that low-elevation hills and mountains (of little more than 1,000 m) in general do not experience a wide enough range of abiotic conditions to promote the evolution of an elevationally-stratified beetle fauna. Area-effects may also be important since the amount of forest at say 1,000 m on a 1,100 m high mountain is generally much smaller than the amount of forest at 1,000 m on a 3,000 m high mountain. Further studies on low mountains in other tropical and temperate locations are needed to test this hypothesis.

Seasonality patterns for most individual beetle species differed among elevations and transects, and there was little consistency in the timing of temporal peaks for species that were common to more than one site. These results suggest that there is considerable plasticity in the temporal dynamics of beetle species in the Wet Tropics. It is unlikely then that the seasonality of many species is driven by factors such as day length or even temperature, which vary little from year to year (but see^15^). Rather, seasonality seems more likely to be driven by factors that display greater temporal and spatial heterogeneity in abundance or availability, such as food resources^13^. For example, the seasonality of herbivorous Chrysomelidae is possibly driven by the availability of new leaves, since many chrysomelids peaked in abundance around the end of the dry-season (Sept-Dec), which coincides with regional peaks in the production of new leaves^47^. A similar pattern of peak abundance and species diversity in the late dry season was also found for chrysomelids in oak-pine forest in NE Mexico^20^. The authors speculate that this pattern may represent the emergence of adults prior to reproduction in the early wet season, when new leaves are more plentiful for growing larvae.

Despite the variability of seasonality of individual species at different sites, some consistent community-level differences in temporal abundance patterns indicate that climatic variables may still influence these beetle assemblages. For instance, the greater seasonality exhibited by species on the Paluma transect could be due to the more severe dry-season experienced at Paluma compared to Atherton. Each elevation at Paluma experienced a much greater proportion of dry-season days with no rainfall (60.7–62.1% of days between Apr 1 and Oct 31) than at Atherton (39.7–49.5% of days; see Table S1). We suggest that while rainfall was a relatively poor predictor of beetle abundance and species richness patterns, moisture availability may ultimately dictate when most species can be active. For instance, Wagner^24^ showed that seasonality of beetles was less marked in a moist swamp-forest than in adjacent primary and secondary forest. Feasibly, once a minimum moisture threshold has been reached, other abiotic and biotic factors, such as temperature or resource availability, may become key drivers governing adult activity levels. In this way, rainfall, temperature, and/or resource availability may interact to affect temporal changes in abundance and species richness.

Our finding that overall abundances and species richness peak in the summer wet-season are consistent with some other studies of insect seasonality in north Queensland rainforests^16,17^, and rainforests from other tropical locations^13,48^. The notable exception is a four year study of the seasonality of beetles from a lowland rainforest site ~300 km north of our Atherton transect^18^. They showed that most beetle species, including those from most of our focal seven (sub)families, peaked in abundance at the end of the dry-season (September to November), then reached a low in the mid-late wet-season (March/April). We can only speculate as to why this discrepancy may occur. It is possible that the sites vary in the seasonality of climatic variables that in turn alter the seasonality of beetles. In particular, Grimbacher and Stork^18^ showed a negative correlation between beetle abundance and rainfall, while we found only positive effects. It is possible that the much greater rainfall at their study site (mean annual rainfall 3,926 mm)^49^ actually supressed beetle activity in the summer wet-season. Clearly much more work is needed to describe regional or global variation in seasonality patterns, and elucidate the abiotic and biotic forces driving seasonality in tropical rainforest insects.

Based on their seasonality and elevational patterns, we tentatively conclude that these groups of beetles are probably not as vulnerable to increased temperatures and longer dry-season length and severity resulting from moderate climate change as many other plant and animal groups, both in the Australian Wet Tropics and potentially around the world. Most species exhibited very long season-lengths, and seasonal changes in abundance are possibly driven by combinations of abiotic and biotic factors, rather than single overarching climatic variables. However, the more marked seasonality among beetles from the drier Paluma transect suggests that if dry-seasons get longer or more severe, as predicted^6^, we should expect seasonality to become more marked in those forests. But since there appears to be considerable plasticity in seasonality patterns of most species, the extinction risk is probably relatively low. Furthermore, García-Robledo *et al*.^19^ found that tropical insects species with wide elevational ranges displayed greater resilience to high temperatures than species restricted to narrow elevational ranges. Given that most of the common species we collected occurred over a wide elevational range, these species are likely to be relatively resilient to higher temperatures.

While our dataset is large, and our results are consistent, the possibility remains that we failed to detect support for our hypothesis due to some other factor. For instance, the lack of marked differences in seasonality with elevation may have been influenced by our choice of target families, which contained many species that were relatively insensitive to elevation. Widespread species are likely more plastic in their responses to environmental variation, while gene-flow may prevent adaptation to local abiotic conditions. By contrast, many flightless beetles in the Wet Tropics display narrow elevational ranges and are restricted to the tops of mountains^6^. However, the flightless mountain-top beetle fauna is a highly specialised, and relatively small, group of insects, and thus less likely to be representative of insects in general than the species from our focal families, which were phylogenetically and ecologically diverse. We therefore suggest that our results may be generally applicable to most winged insect groups on hills and low mountains in the Australian Wet Tropics, and also around the world.

## Electronic supplementary material

Supplementary Information
