# Supplementary material for: Seasonal variation in a diverse beetle assemblage along two elevational gradients in the Australian Wet Tropics
Source: Sci Rep. 2018 Jun 4;8:8559. doi: 10.1038/s41598-018-26216-8 (PMC5986770; doi:10.1038/s41598-018-26216-8)
Supplement: Supplementary file 1 — Supplementary Information [file 41598_2018_26216_MOESM1_ESM.doc]

**Supporting materials**

**Seasonal variation in a diverse beetle assemblage along two elevational gradients in the Australian Wet Tropics**

C. W. Wardhaugh, M. Stone, and N.E. Stork

a) Atherton

b) Paluma

**Figure S1.** Boxplots of total abundance and species diversity with season (N= 4) and elevation (N= 5) at the a) Atherton and b) Paluma transects.

Seasonal abundances at each elevation for each family Atherton


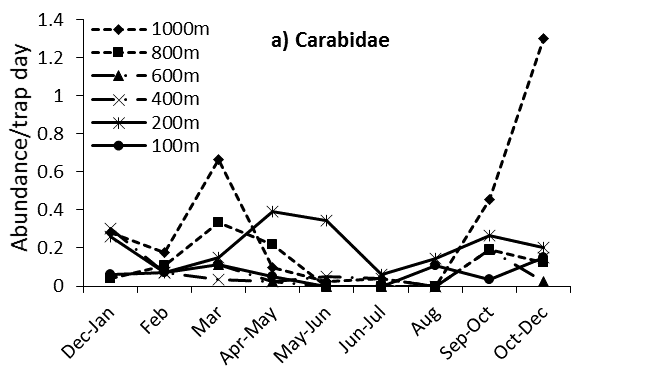

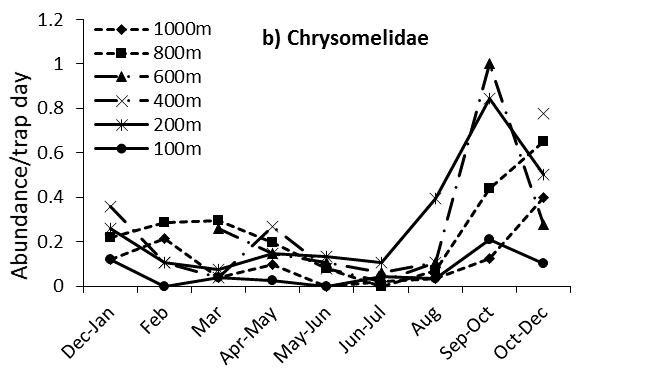

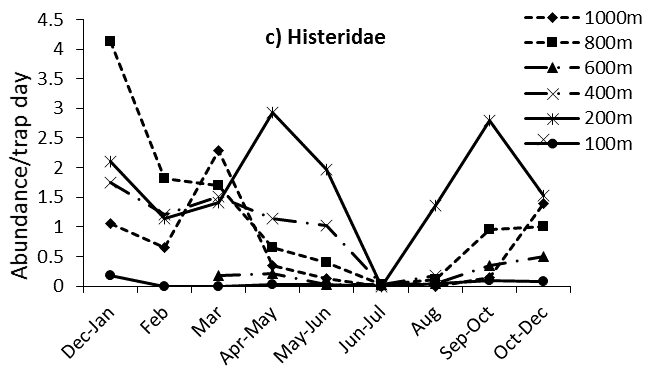

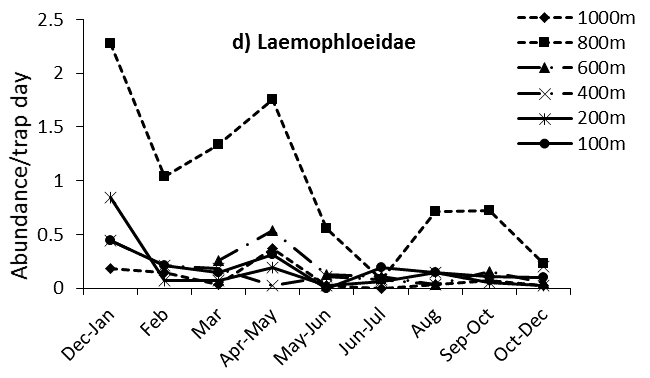

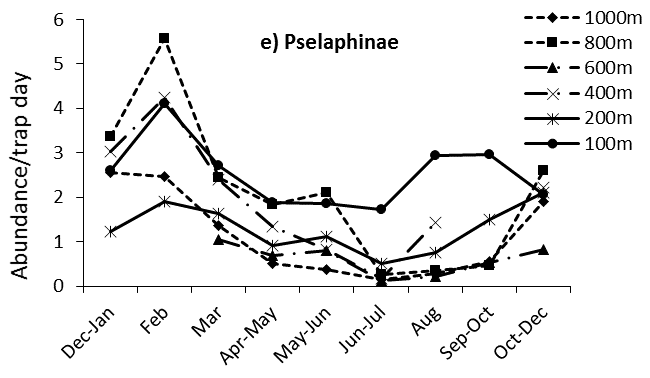

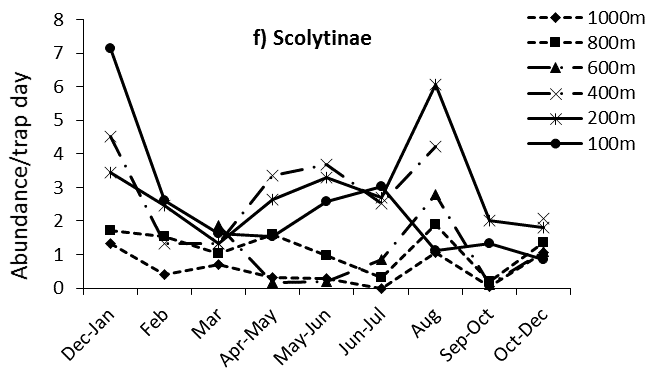

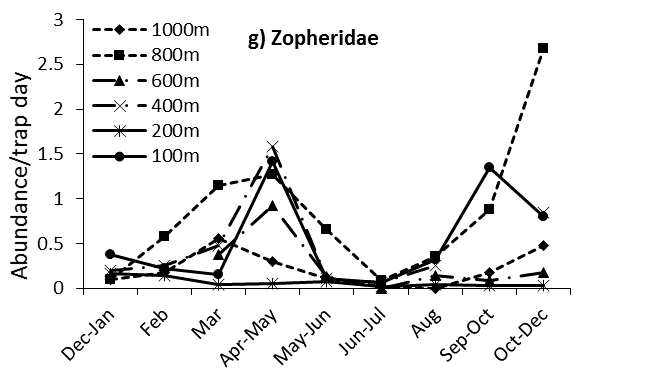


**Figure S2**. Seasonal variation in abundance/trap day at each elevation for each family at Atherton. Note that the temporal axes are not to scale.

Seasonal abundances at each elevation for each family Paluma


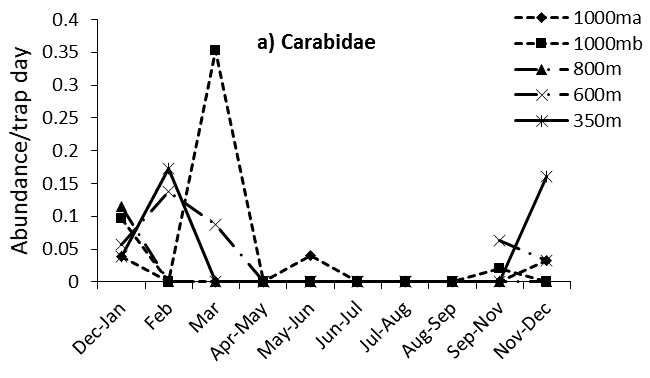

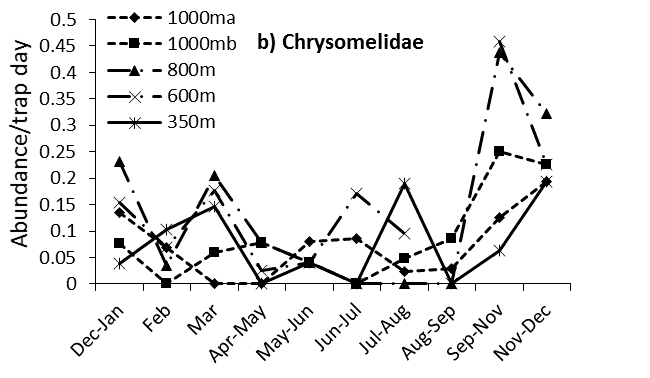

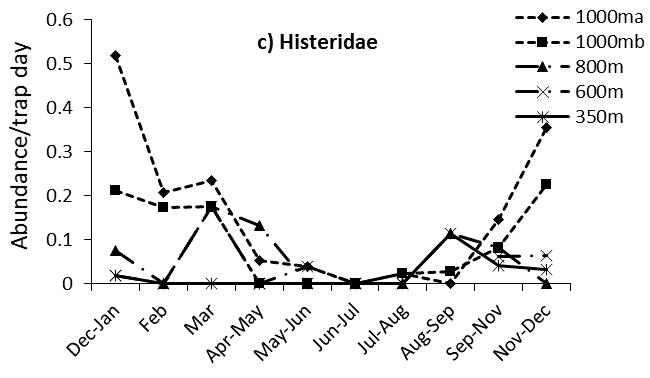

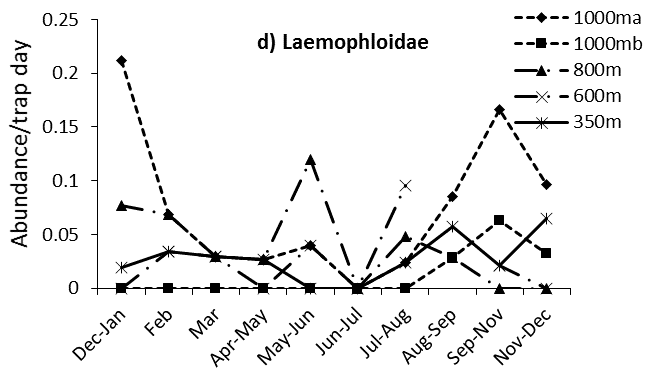

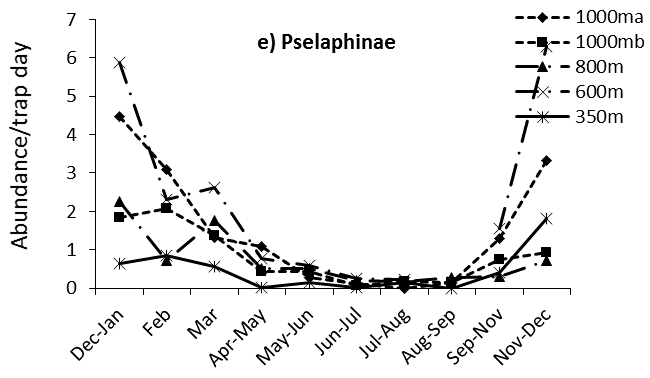

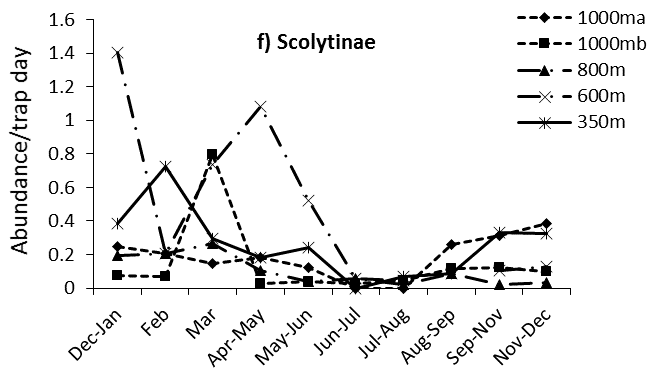

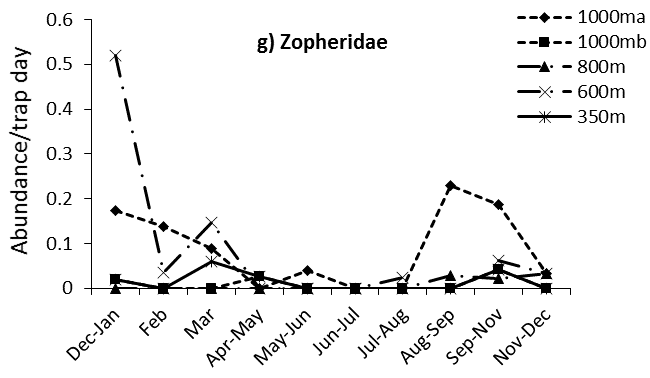


**Figure S3**. Seasonal variation in abundance/trap day at each elevation for each family at Paluma. Note that the temporal axes are not to scale.


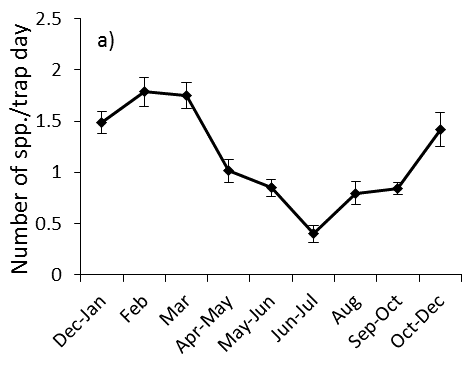

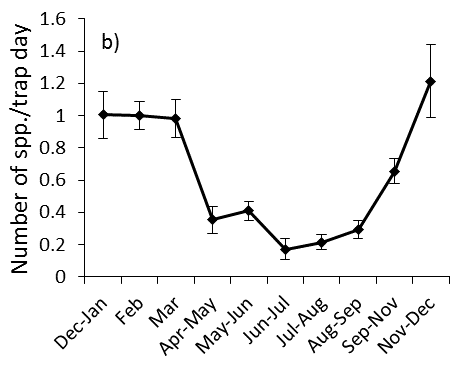


**Figure S4**. Seasonal variation in the mean number of species (± SE) collected from all seven focal families combined per trap day across all elevations on a) the Atherton transect, and b) the Paluma transect. Note that the temporal axes are not to scale.

Sp diversity at each elevation for each family Atherton


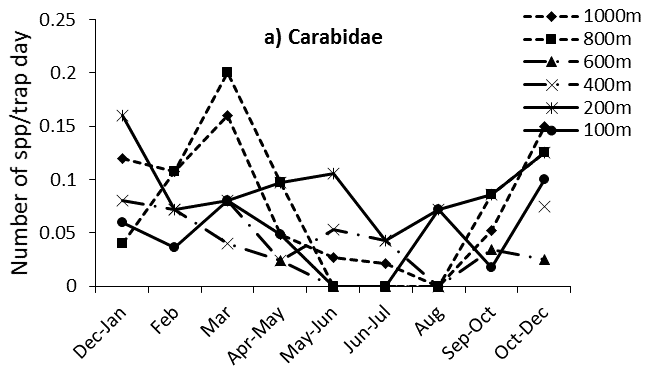

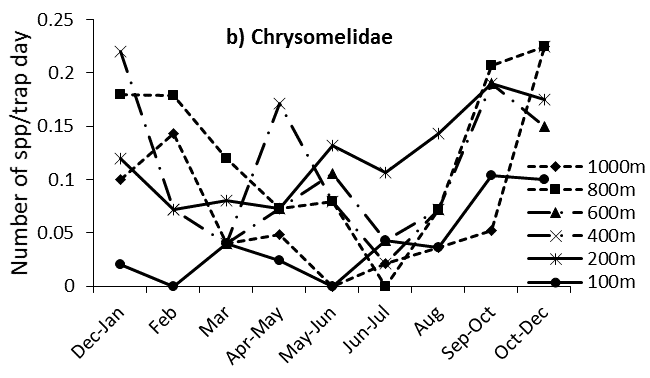

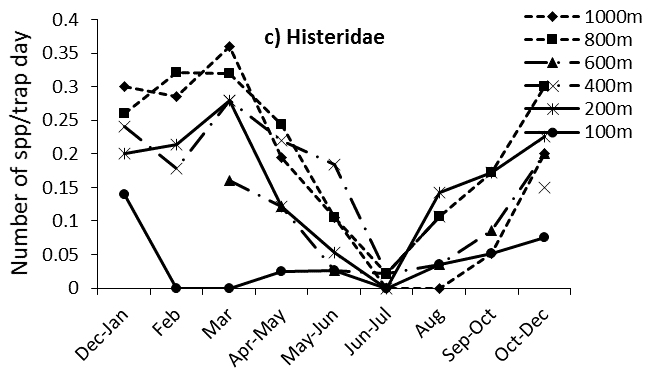

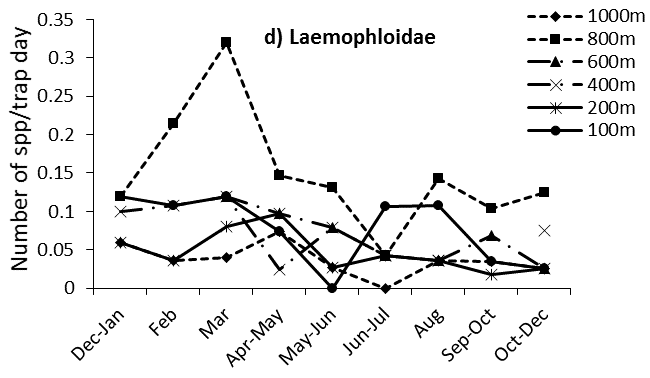

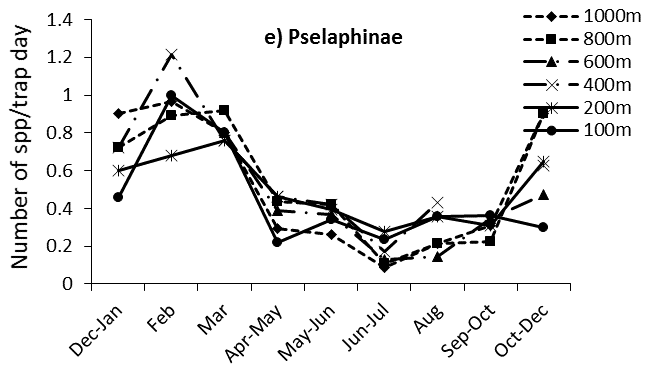

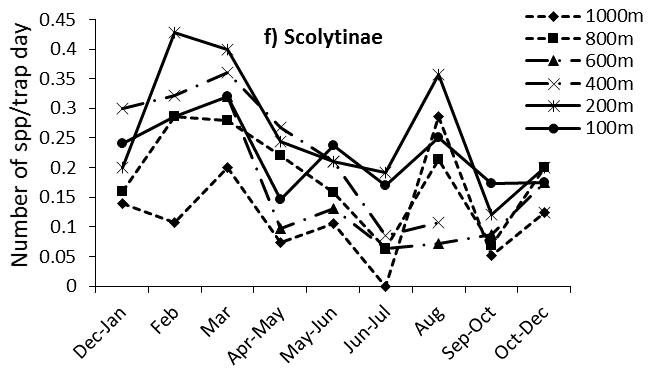

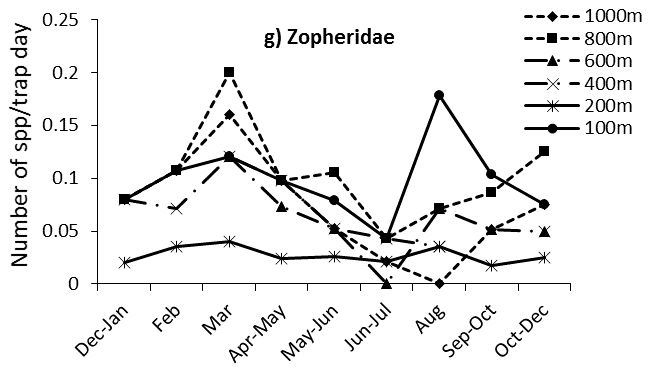


**Figure S5**. Seasonality of species diversity for each family at each elevation at Atherton. Note that the temporal axes are not to scale.

Sp diversity at each elevation for each family Paluma


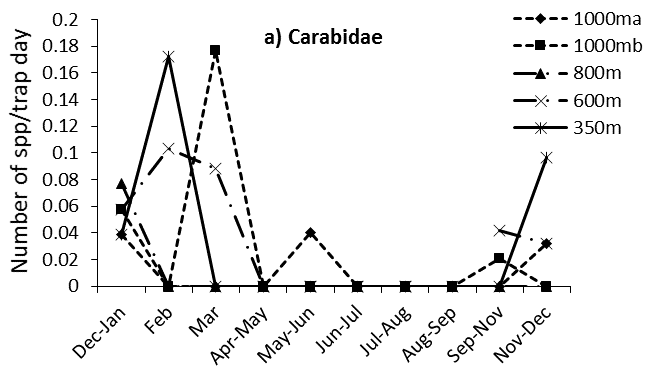

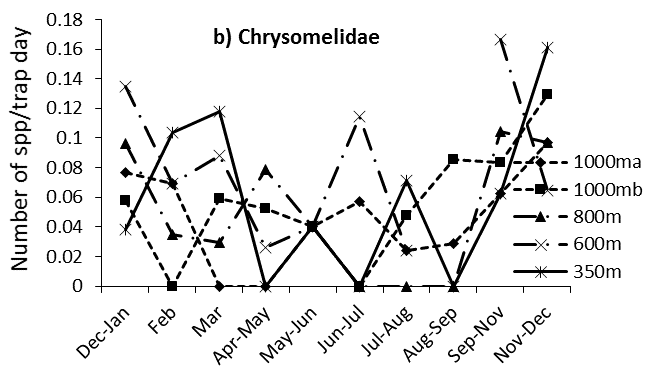

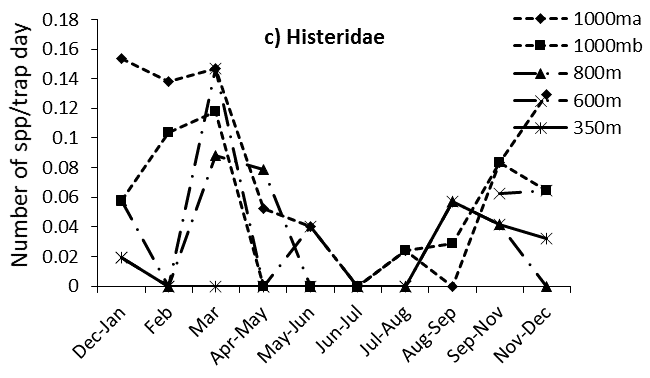

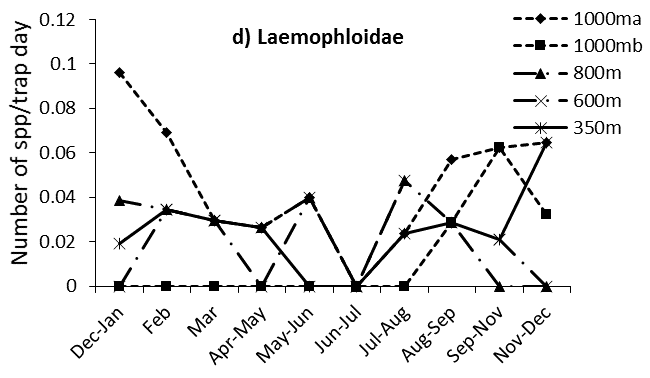

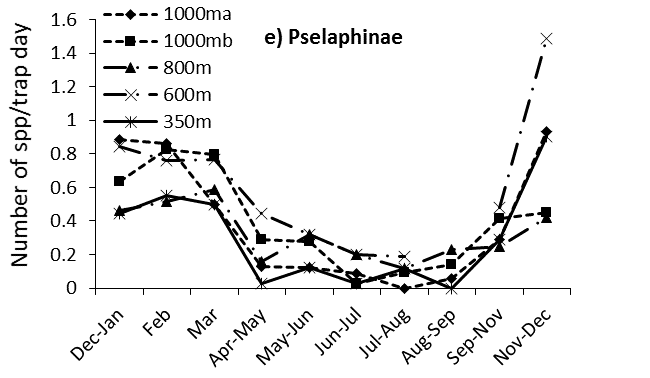

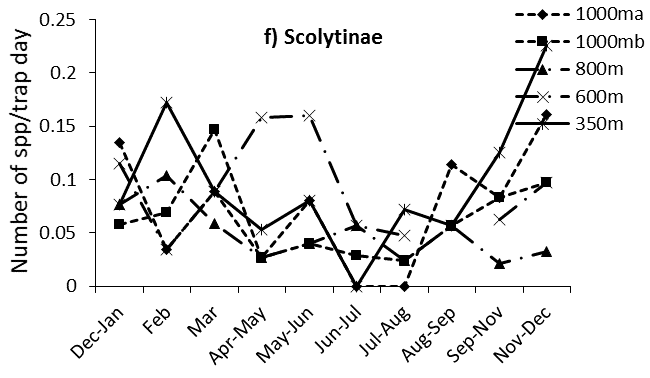

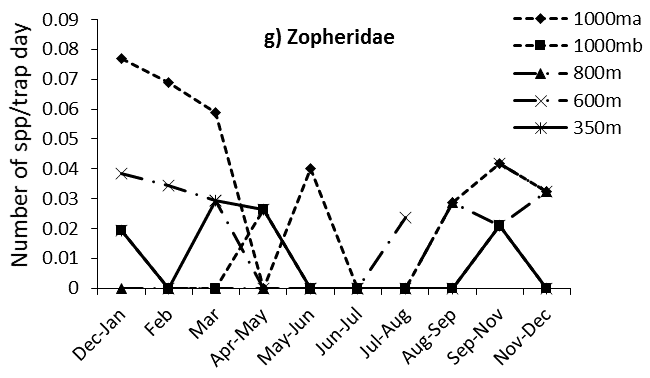


**Figure S6**. Seasonality of species diversity for each family at each elevation at Paluma. Note that the temporal axes are not to scale.

**Table S1**. Location and characteristics for each site sampled along both altitudinal transects. Shown are forest type, mean annual precipitation (MAP) from 1990 to 2014, actual recorded rainfall during the study (2007), and rainfall seasonality (DS0MM, the percentage of dry season days (between Apr 1st and Oct 31^st^) with 0mm of rain).

| Transect | Elevation (m) | Lat. | Long. | Forest type^a^ | MAP (mm) | Rainfall (mm) | DS0MM (%) |
| --- | --- | --- | --- | --- | --- | --- | --- |
| Atherton | 100 | -17.72 | 145.86 | CMVF | 3204 | 3235 | 43.9 |
|  | 200 | -17.66 | 145.87 | CMVF | 3254 | 3286 | 42.1 |
|  | 400 | -17.61 | 145.77 | CMVF | 2924 | 2960 | 39.7 |
|  | 600 | -17.67 | 145.71 | CMVF | 2698 | 2660 | 40.7 |
|  | 800 | -17.60 | 145.63 | NVF | 2746 | 2644 | 40.7 |
|  | 1000 | -17.70 | 145.52 | NVF | 1603 | 1449 | 49.5 |
| Paluma | 350 | -19.01 | 146.27 | NVF | 1519 | 1724 | 61.2 |
|  | 600 | -19.00 | 146.24 | NVF | 1519 | 1724 | 61.2 |
|  | 800 | -19.01 | 146.22 | NVF | 1653 | 1843 | 62.1 |
|  | 1000 (a) | -19.02 | 146.16 | ACF | 1840 | 2020 | 60.7 |
|  | 1000 (b) | -19.00 | 146.21 | ACF | 1653 | 1843 | 62.1 |

**Table S2**. Linear mixed effects fit by REML model selection results. The fixed variables include the predictor variables elevation (N= 5) and season (N=4) and climatic co-variates of rainfall and maximum and minimum temperature. AIC’s in bold are the best model fits for each respective beetle attribute and were used in further analyses.

|  | **Atherton** | | | **Paluma** | | |
| --- | --- | --- | --- | --- | --- | --- |
| **Fixed variables included in the model** | Total abund. | Abund. of 7 focal Fam. | Spp. rich of 7 focal Fam. | Total abund. | Abund. of 7 focal Fam. | Spp. rich of 7 focal Fam. |
| **Elevation, Season, Rainfall, Tmax, Tmin** | **445** | 186 | 60 | **426** | 171 | 51 |
| **Elevation, Season, Rainfall, Tmax** | 451 | 185 | 56 | 429 | 170 | 49 |
| **Elevation, Season, Rainfall, Tmin** | 452 | 185 | 57 | 430 | 167 | 46 |
| **Elevation, Season, Tmax, Tmin** | 447 | 184 | 54 | 428 | 165 | 42 |
| **Elevation, Season** | 464 | 189 | 73 | 438 | 174 | 60 |
| **Elevation, Season, Rainfall** | 462 | 191 | 79 | 431 | 180 | 68 |
| **Elevation, Season, Tmax** | 453 | **182** | 55 | 435 | **163** | 44 |
| **Elevation, Season, Tmin** | 454 | 187 | **49** | 441 | 164 | **36** |

**Table S3**. Post-hoc Tukey pair-wise test results with Bonferroni-Holm corrections for multiple comparisons for elevation and season at a) Atherton and b) Paluma. Significant P-values are in bold.

a) Atherton

| **Variable** | **Pair-wise test** | **Total abund** | | **Focal Fam abund** | | **Spp. diversity** | |
| --- | --- | --- | --- | --- | --- | --- | --- |
|  |  | z | p | z | p | z | p |
| **Elevation** | 800-100 | 0.98 | 1.00 | 1.08 | 1.00 | **5.14** | **<0.0001** |
|  | 1000-100 | 2.16 | 0.31 | -0.35 | 1.00 | **4.22** | **0.0002** |
|  | 800-200 | 0.88 | 1.00 | 0.77 | 1.00 | **3.81** | **0.001** |
|  | 1000-200 | 2.07 | 0.35 | -0.67 | 1.00 | **3.00** | **0.02** |
|  | 400-100 | 0.44 | 1.00 | 0.71 | 1.00 | **2.80** | **0.03** |
|  | 800-400 | 0.53 | 1.00 | 0.37 | 1.00 | 2.52 | 0.06 |
|  | 1000-400 | 1.73 | 0.67 | -1.05 | 1.00 | 1.83 | 0.27 |
|  | 200-100 | 0.09 | 1.00 | 0.32 | 1.00 | 1.41 | 0.46 |
|  | 1000-800 | 1.21 | 1.00 | -1.47 | 1.00 | -0.57 | 0.56 |
|  | 400-200 | 0.35 | 1.00 | 0.40 | 1.00 | 1.43 | 1.43 |
| **Season** | Winter-Autumn | 0.33 | 1.00 | 0.77 | 1.00 | 2.44 | 0.09 |
|  | Winter-Spring | 0.44 | 1.00 | 1.02 | 1.00 | 1.96 | 0.25 |
|  | Winter-Summer | 0.38 | 1.00 | -0.40 | 1.00 | 1.91 | 0.25 |
|  | Spring-Autumn | -0.31 | 1.00 | -0.70 | 1.00 | 0.82 | 1.00 |
|  | Summer-Autumn | -0.23 | 1.00 | 1.72 | 0.42 | -0.11 | 1.00 |
|  | Summer-Spring | 0.07 | 1.00 | 2.40 | 0.10 | -0.74 | 1.00 |

b) Paluma

| **Variable** | **Pair-wise** | **Total abund** | | **Focal Fam abund** | | **Spp. diversity** | |
| --- | --- | --- | --- | --- | --- | --- | --- |
|  |  | z | p | z | p | z | p |
| **Elevation** | 1000-350 | **3.89** | **0.0006** | **3.93** | **0.0005** | **3.35** | **0.005** |
|  | 1000-600 | 2.39 | 0.08 | **2.61** | **0.04** | 1.90 | 0.29 |
|  | 800-350 | 2.15 | 0.13 | 2.21 | 0.11 | 1.72 | 0.34 |
|  | 1000-800 | 1.71 | 0.26 | 1.67 | 0.29 | 1.51 | 0.39 |
|  | 600-350 | 1.20 | 0.46 | 1.09 | 0.54 | 1.26 | 0.41 |
|  | 800-600 | 0.91 | 0.46 | 1.11 | 0.54 | 0.45 | 0.65 |
| **Season** | Spring-Autumn | 1.73 | 0.33 | 1.79 | 0.37 | 3.04 | 0.01 |
|  | Winter-Autumn | 2.05 | 0.24 | 2.04 | 0.25 | 1.4 | 0.79 |
|  | Summer-Autumn | 1.87 | 0.31 | 0.80 | 1.00 | 1.24 | 0.82 |
|  | Summer-Spring | 0.39 | 1.00 | -0.77 | 1.00 | -1.27 | 0.82 |
|  | Winter-Spring | 0.70 | 1.00 | 0.96 | 1.00 | -0.19 | 1.00 |
|  | Winter-Summer | 0.34 | 1.00 | 1.22 | 0.89 | 0.55 | 1.00 |

**Table S4**. The spread of monthly peak abundances for the 36 species where at least 10 individuals were collected from both transects.

| Peak abundance | No. species |
| --- | --- |
| Same month | 4 |
| Across 2 months | 12 |
| Across 3 months | 4 |
| Across 4 months | 4 |
| Across 5 months | 1 |
| Aseasonal on one transect | 9 |
| Aseasonal on both transects | 2 |

**Table S5**. The spread of monthly peak abundances for the 58 species where at least 10 individuals were collected from more than one site. Most species were aseasonal on at least one site, while most of the remaining species (18/29) peaked with a three month period across all sites.

| Peak abundance | No. of species |
| --- | --- |
| Same month | 6 |
| Across 2 months | 6 |
| Across 3 months | 6 |
| Across 4 months | 3 |
| Across 5 months | 4 |
| Across 6 months | 2 |
| Across 7 months | 2 |
| Aseasonal at one or more sites | 25 |
| Aseasonal at all sites | 4 |
